# Corona-Krise: Staatsbeteiligungen bleiben eine problematische Intervention in den Markt

**DOI:** 10.1007/s10273-020-2802-4

**Published:** 2020-12-26

**Authors:** Klaus-Heiner Röhl, Christian Rusche

**Affiliations:** 1 Kompetenzfeld Digitalisierung, Strukturwandel und Wettbewerb, Institut der deutschen Wirtschaft Köln e.V., Konrad-Adenauer-Ufer 21, 50668 Köln, Deutschland; 2 Institut der deutschen Wirtschaft Köln e.V., Konrad-Adenauer-Ufer 21, 50668 Köln, Deutschland

**Keywords:** E61, H12, O25

## Abstract

Die Corona-Pandemie erfordert staatliche Eingriffe, um negative ökonomische Auswirkungen auf Konsumenten und Unternehmen abzumildern. Es bestehen jedoch auch Gefahren bei Interventionen durch den Staat: So können ineffiziente Strukturen konserviert werden, Märkte verzerrt und langfristig negative Struktureffekte ausgelöst werden. Deswegen ist es wichtig, dass der Staat bei seinen Interventionen auf Marktkonformität achtet. Das bedeutet, dass keine Strukturen geschaffen werden dürfen, die sich nicht auch am Markt hätten bilden können oder am Wettbewerbsrecht gescheitert wären. Staatliche Eingriffe sollten überdies nicht dazu genutzt werden, um politischen Einfluss auszuüben.

## Abstract (en)

The Coronavirus pandemic has presented society with major challenges that have required state intervention.. While government measures are necessary in an external crisis such as the current pandemic, negative effects can also arise from excessive government intervention, particularly for the economy. An analysis of the OECD countries shows that the state already played a major role in the economies of many countries before the coronavirus crisis, especially in Germany. However, state participation in companies does not necessarily have to lead to negative effects if it is structured appropriately. This article discusses how government measures, especially the extension of public ownership, should be designed in order to maintain competition and use the free market as an allocation instrument where possible. This means that no market structures should be created which could not have been created or which fail to comply with competition law. Moreover, state intervention should not be used to exert political influence on business decisions.

Die Corona-Pandemie ist ein exogener Schock für die Weltwirtschaft und die einzelnen Volkswirtschaften, welcher das öffentliche Leben in einer in Friedenszeiten bislang unbekannten Weise beeinträchtigt. Die international stark eingebundene deutsche Volkswirtschaft wurde hart getroffen. Vor dem Hintergrund eines solchen externen Schocks sind staatliche Wirtschaftseingriffe zur Verringerung von Unsicherheit und der Sicherung von Unternehmen sowie Arbeitsplätzen gerechtfertigt. Diese staatlichen Maßnahmen müssen jedoch so ausgestaltet werden, dass negative Auswirkungen wie höhere Preise, geringere Innovationstätigkeit und Wettbewerbsverzerrungen vermieden werden. Besonders problematisch erscheinen direkte Staatsbeteiligungen an Unternehmen, die den Markt dauerhaft verzerren können. Sie bedürfen deshalb klarer Regeln.

Die Pandemie hat die Position des Staates in Deutschland tendenziell gestärkt. Staatliche Akteure führten Ausgangs- sowie Kontaktbeschränkungen ein, verfügten Quarantänemaßnahmen und Grenzschließungen. Der Staat setzte im Lockdown Betriebsschließungen durch und muss es angesichts steigender Fallzahlen nun wieder tun. Zahlreiche Unternehmen wurden und werden durch die Maßnahmen zur Eindämmung der Pandemie sowie durch die Umsatzeinbrüche infolge von Nachfrageeinbrüchen und Produktionsschwierigkeiten vor erhebliche Herausforderungen gestellt, was die Versorgung der Gesellschaft zum Teil erschwert hat. In der Folge kam es zu zunehmenden staatlichen Eingriffen in das Wirtschaftsgeschehen, beispielsweise durch direkte Beteiligungen an der Deutschen Lufthansa AG, der TUI Group und CureVac. Zudem wurden und werden umfangreiche staatliche Garantien, Zuschüsse sowie Kredite gewährt. Bei einem möglichen Ausbleiben einer weiteren wirtschaftlichen Erholung infolge einer zweiten Infektionswelle dürften staatliche Eingriffe nochmals zunehmen.

## Der Staat muss handeln

Eine wirtschaftliche Krise ist jedoch nicht nur eine Herausforderung, sondern kann auch eine Chance darstellen. Bereits 1934 formulierte Joseph Schumpeter die These, dass die wirtschaftliche Entwicklung nicht geradlinig erfolgt. Ökonomische Krisen gehören fest zum Entwicklungsprozess einer Volkswirtschaft, weil sie Innovationen zum Durchbruch verhelfen und die Grundlagen für einen erneuten Aufschwung bilden. Prägnanter formulierte es der ehemalige US-Finanzminister Andrew Mellon gegen-

**Dr. Klaus-Heiner Röhl** ist Senior Economist beim Institut der deutschen Wirtschaft am Standort Berlin.

**Dr. Christian Rusche** ist Economist für Industrieökonomik und Wettbewerb beim Institut der deutschen Wirtschaft in Köln.

* Dieser Artikel beruht auf Röhl und Rusche (2020).

über US-Präsident Herbert Hoover (Präsident von 1929 bis 1933): „Values will be adjusted, and enterprising people will pick up the wrecks from less competent people."

Somit können ökonomische Krisen mittelfristig zu überdurchschnittlichen Produktivitätsfortschritten führen, wenn Unternehmen mit neuen Technologien den Platz insolventer, unwirtschaftlicher Unternehmen einnehmen oder Unternehmen neue Technologien einführen sowie ineffiziente Strukturen und Prozesse verbessern, um wettbewerbsfähig zu bleiben. Davon profitieren auch die Konsumenten, da Unternehmen aus dem Markt ausscheiden, welche die Bedürfnisse der Kunden nicht adäquat befriedigen konnten. Gerade während einer Krise ist der Wettbewerbsdruck stärker als während eines Aufschwungs, weil Mittel knapper bemessen sind, Erwartungen sich eintrüben und Konsumenten sowie Unternehmen ihre Ausgaben stärker überprüfen. Im Sinne von Schumpeter handelt es sich um eine kreative Zerstörung, bei der die Krise als Katalysator wirkt.

Diese Sichtweise ist jedoch umstritten. So schrieb US-Präsident Herbert Hoover in seinen Memoiren zur großen Depression: „Panic had always left a trail of unnecessary bankruptcies which injured the productive forces of the country." Somit kann es in einer großen Krise für den Staat gefährlich sein, nicht stützend einzugreifen: Falls jedes Unternehmen jederzeit insolvent sein kann, vertrauen sich die Wirtschaftsakteure gegenseitig nicht mehr. Durch diese Entwicklungen steigt die Zahl an Insolvenzen immer weiter an, wodurch die Wirtschaftsakteure noch vorsichtiger werden und sich die Krise selbst verstärkt. Durch die Liquidation von Unternehmen mit funktionierendem Geschäftsmodell kann die Krise auch die Grundlage für späteres Wachstum zerstören, weil zu viele Unternehmen und ihre Anlagen liquidiert werden. Auch Start-ups erhalten dann keine Finanzierung mehr und können somit mittel- bis langfristig nicht den Platz insolventer Unternehmen einnehmen. Ein Eingriff des Staates kann in einer solchen Situation Vertrauen wiederherstellen oder sichern, die Nachfrage stabilisieren und die Krise abmildern.

Zudem hatte die wirtschaftliche Kontraktion infolge der Corona-Pandemie keine strukturellen, wirtschaftlichen oder regulatorischen Ursachen. Der Auslöser der Krise war keine wirtschaftliche Übertreibung oder das Handeln von „incompetent people" wie Mellon es beschreibt. Zwar verstärkt die Wirtschaftskrise den Strukturwandel in einigen Bereichen – beispielsweise in Richtung Digitalisierung –, doch sie gefährdet auch viele Unternehmen mit einem prinzipiell funktionstüchtigen Geschäftsmodell. Idealerweise sollte der Staat hier so eingreifen, dass die kreative Zerstörung wirken kann, ohne dass gesunde Unternehmen in Mitleidenschaft gezogen und insolvent werden. Eine Insolvenz in jedem Fall zu verhindern, ist daher nicht angebracht, zumal diese auch zur Sanierung eines Unternehmens dienen kann. Die Unterscheidung, welches Unternehmen gerettet wird und welches nicht, ist jedoch nicht trivial und unter Umständen mit Härten verbunden (z. B. mit Arbeitsplatzverlusten). Zudem kann ein selektives Eingreifen zu den gleichen Krisenwirkungen führen, die bereits beschrieben wurden: Im Prinzip kann weiterhin jedes Unternehmen insolvent werden. Ein zu weitreichendes Eingreifen kann die staatlichen Möglichkeiten dagegen überfordern und den Staat in die Handlungsunfähigkeit führen. Dennoch erscheinen staatliche Eingriffe in einer akuten Krise historischen Ausmaßes als notwendig.

## Der Staat im Wirtschaftsgeschehen

Mit einem staatlichen Wirtschaftseingriff gehen jedoch auch negative Effekte einher. So kann die Ausschaltung des Wettbewerbs durch konservierende Staatseingriffe Produktivitätsfortschritte verhindern, weil beispielsweise nicht wettbewerbsfähige „Zombieunternehmen" am Markt gehalten werden. Dies ist umso stärker der Fall, wenn sich Erwartungen aufbauen, dass der Staat auch in weiteren Krisen zugunsten etablierter, aber ineffizienter Unternehmen eingreift. Die Durchsetzung von Innovationen wird so behindert oder gar verhindert. Zur Beurteilung, ob und in welchem Umfang ein Staat eingreifen sollte, ist eine Analyse hilfreich, in welchem Umfang der Staat bereits als Akteur vertreten ist und welche Erfahrungen in anderen Staaten gesammelt wurden. Dadurch können verschiedene Modelle der staatlichen Intervention miteinander verglichen und Schlussfolgerungen über die Wirkung dieser Eingriffe gezogen werden.

Die OECD misst alle fünf Jahre mithilfe ihrer Product-Market-Regulation-Untersuchung (PMR), welche Politikmaßnahmen den Wettbewerb auf Produktmärkten stärken oder behindern. In diesem Zusammenhang wird auch die Rolle staatseigener Unternehmen beleuchtet. Aus der letzten Untersuchung aus dem Jahr 2018 liegen Daten für 36 der 37 OECD-Mitgliedstaaten vor (die USA fehlen). Dabei variiert die Reichweite staatseigener Unternehmen sehr stark. Ein staatseigenes Unternehmen ist definiert als „jede juristische Person, die nach innerstaatlichem Recht als Unternehmen gilt und in der der Staat eine Eigentümerfunktion wahrnimmt".

Der PMR-Indikator „Reichweite von staatseigenen Unternehmen" ist im Intervall von 0 bis 6 definiert. Insgesamt werden 25 Sektoren betrachtet. Darunter befinden sich sieben Netzwerksektoren (z. B. Lufttransport, Gas, Elektrizität), welche ein Gewicht von jeweils 1/8 bekommen. 18 weitere Sektoren (z. B. Glücksspiel, Großhandel und Bau)

bekommen zusammen ebenfalls ein Gewicht von 1/8; somit hat jeder dieser 18 Wirtschaftsbereiche ein Gewicht von 1/144. Ist in einem betrachteten Sektor ein staatseigenes Unternehmen aktiv, bekommt dieser Bereich den Wert 6, ansonsten ist der Wert 0. Der Gesamtwert für ein Land setzt sich aus der Summe der Sektorwerte multipliziert mit dem jeweiligen Gewicht zusammen. Ein Wert von 6 für einen Staat bedeutet folglich, dass in jedem der betrachteten Wirtschaftsbereiche mindestens ein staatseigenes Unternehmen aktiv ist. Ein Wert von Null bedeutet, dass es in den genannten Sektoren keine staatseigenen Unternehmen gibt.

Innerhalb der OECD (ohne USA) weist Chile mit 1,13 den geringsten Wert und Frankreich mit 5,38 mit Abstand vor Polen mit 4,75 den höchsten Wert auf. Deutschland liegt mit 4,27 auf Platz 5. Somit gehört Deutschland zu den Staaten, in denen staatseigene Unternehmen in vielen Wirtschaftsbereichen vertreten sind. Dies ist vor allem auf die starke wirtschaftliche Betätigung der Kommunalebene zurückzuführen.

### Kommunale Ebene in Deutschland stark in der Wirtschaft vertreten

Die kommunale Wirtschaftsaktivität in Deutschland hat eine lange Tradition. Während klassischerweise Bereiche der Daseinsvorsorge durch kommunale Betätigung gekennzeichnet waren – hierzu zählten neben der Wasserversorgung sowie Abwasser- und Müllentsorgung früher auch die netzgebundene Energieversorgung mit Strom und Gas sowie (als gesamtstaatliche Aufgabe) die Telekommunikation – haben die Kommunen im Laufe der Zeit ihre Betätigung auf viele weitere Bereiche ausgedehnt.

Nach einer Phase der Privatisierung kommunaler Tätigkeitsfelder von Ende der 1980er Jahre bis kurz nach der Jahrtausendwende ist die kommunale Wirtschaftstätigkeit seither wieder im Aufschwung. Städte und Gemeinden setzen auf öffentliche Angebote von Dienstleistungen und nutzen ihre Vorteile – Begünstigung bei der Mehrwertsteuer, Möglichkeit zur Quersubventionierung zwischen rentablen und verlustbringenden Bereichen, Ausnahmen vom Kartellrecht und Nähe zur Politik – zum Schaden des Wettbewerbs aus. Die Zahl der Eigenbetriebe sowie der privatwirtschaftlich organisierten Unternehmen im Eigentum der öffentlichen Hand wächst seit Jahren kräftig. Die inzwischen mehr als 15.800 kommunalen Unternehmen entfallen definitionsgemäß fast ausschließlich auf die Flächenländer, die drei Stadtstaaten wiesen 2017 weitere 774 Unternehmen auf. Nach Angaben des Statistischen Bundesamts stieg die Zahl der kommunalen Unternehmen von 2000 bis 2017 um 45 %. Ihre Umsätze wuchsen im gleichen Zeitraum sogar um 141 %, während die Lieferungen und Leistungen umsatzsteuerpflichtiger Unternehmen insgesamt in Deutschland nur um 59 % zulegten.

### Ausgelöste Verzerrung maßgeblich

Die Verbreitung staatseigener Unternehmen sagt jedoch nicht direkt etwas über die Wirkung auf die Wirtschaft und mögliche Verzerrungen des Marktes aus. Dies wird am PMR-Subindex „Distortions Induced by State Involvement“ deutlich. Dieser misst die vom Staat ausgelösten wirtschaftlichen Verzerrungen und vergleicht sie international. Dabei kann die Verzerrung unter anderem vom Eigentümer Staat, dem Staatseingriff über eigene Beschaffung, Preiskontrollen sowie von der Komplexität von Gesetzen und Gesetzgebungsverfahren herrühren. Für die jeweilige Form der ökonomischen Verzerrung verwendet die OECD einen entsprechenden Indikator wie den bereits angesprochenen Indikator „Reichweite von staatseigenen Unternehmen“.

Die Verzerrungen werden analog zur Reichweite staatseigener Unternehmen ebenfalls auf dem Intervall von 0 bis 6 gemessen. Null bedeutet in diesem Zusammenhang keinerlei Verzerrungen, während ein Wert von 6 auf große Verzerrungen hindeutet. Mit einem Wert von 0,84 wies das Vereinigte Königreich im Jahr 2018 den niedrigsten Wert aus. Die höchsten Werte verzeichneten Kolumbien mit 2,18 und die Türkei mit 2,20. Deutschland war mit einem Wert von 1,41 im unteren Drittel der 36 OECD-Staaten angesiedelt und weist somit trotz starker wirtschaftlicher Betätigung des Staates – auf kommunaler Ebene – eine vergleichsweise geringe staatliche Verzerrung des Wirtschaftsgeschehens auf.

Welche wirtschaftlichen Auswirkungen eine hohe Verzerrung seitens des Staates haben kann, wird anhand von Abbildung 1 deutlich. Hier sind das Bruttoinlandsprodukt pro Kopf und die vom Staat induzierte wirtschaftliche Verzerrung abgebildet. Wird von der Schweiz, Luxemburg und Irland abgesehen, ergibt sich ein deutlicher fallender Zusammenhang zwischen Verzerrung und BIP pro Kopf. Das heißt, je höher die Verzerrung, desto geringer ist tendenziell die Wirtschaftsleistung pro Kopf und umgekehrt. Der Korrelationskoeffizient auf Basis eigener Berechnungen ohne Schweiz, Irland und Luxemburg, die unter anderem auch aufgrund des Vorhandenseins von Sitzen international tätiger Konzerne über ein außergewöhnlich hohes BIP trotz einer hohen Verzerrung verfügen, beträgt -0,54. Dieser Wert weist auf das Vorhandensein eines negativen Zusammenhangs für die überwiegende Zahl der OECD-Staaten hin. Die Wirkung staatlicher Eingriffe in die Wirtschaft kann sich somit auf den Wohlstand eines Landes auswirken.

Abbildung 1

**BIP pro Kopf und durch Staat verursachte ökonomische Verzerrungen 2018 in den OECD-Staaten[1]**

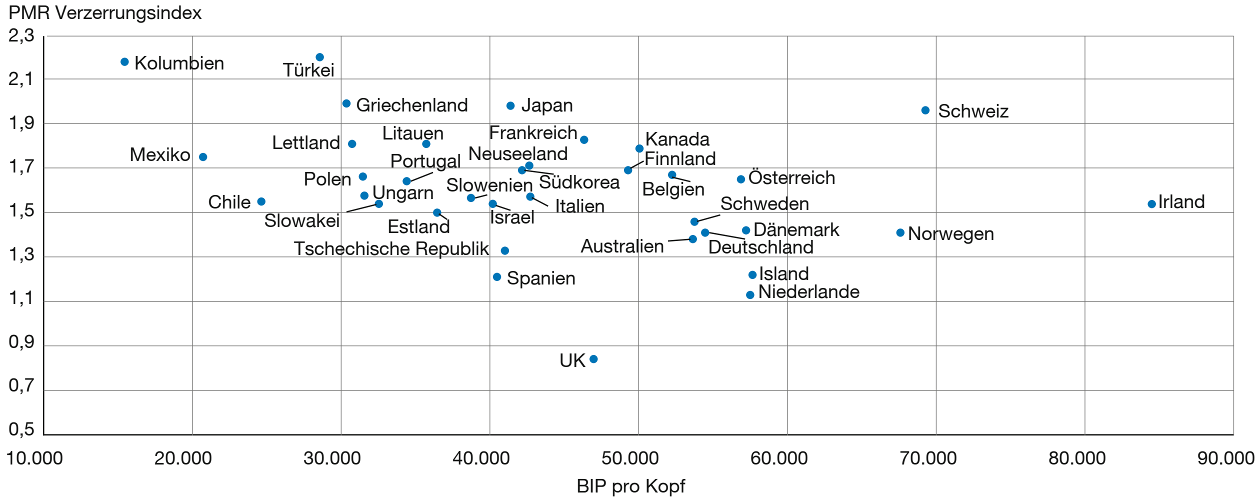

[1] ohne USA und Luxemburg.

Anmerkungen: BIP pro Kopf in US-Dollar, aktuelle Preise und aktuelle Kaufkraftparität; ökonomische Verzerrungen abgebildet durch den PMR-Subindex „Distortions Induced by State Involvement“, Skala des Indikators: 0 bis 6. Ein Wert von 0 bedeutet keine Verzerrung des Wirtschaftsgeschehens und ein Wert von 6 bedeutet eine massive Verzerrung.

Quelle: OECD (2020a, 2020b).

Das bloße Vorhandensein staatlicher Unternehmen in einem Sektor reicht für eine negative Wirkung jedoch nicht aus. So ist für den Indikator Reichweite staatseigener Unternehmen und dem BIP pro Kopf tendenziell kein Zusammenhang erkennbar. Der Korrelationskoeffizient beträgt für alle 36 OECD-Mitgliedstaaten, für die Werte vorlagen, 0,24. Der Korrelationskoeffizient reduziert sich auf 0,1, wenn Luxemburg, die Schweiz und Irland nicht betrachtet werden. Eine staatliche Beteiligung an einem Unternehmen ist somit kein hinreichendes Kriterium für negative wirtschaftliche Auswirkungen. So kommt es insbesondere auch auf den Zweck des staatseigenen Unternehmens (Gewinnmaximierung oder andere Ziele wie Versorgungssicherheit), die Unternehmensführung und das jeweilige regulatorische Umfeld an.

Zur Vermeidung einer möglichen Verzerrung gilt es, die zwei ordnungspolitischen Grundsätze der Sozialen Marktwirtschaft bei Staatseingriffen zu beachten:

1. Die Koordination der wirtschaftlichen Handlungen soll über Märkte durch Preise und Wettbewerb erfolgen, was Privateigentum, Vertragsfreiheit und Haftung erfordert.

2. Die Marktprozesse sollen durch den Einsatz der wirtschaftspolitischen Instrumente nicht gestört werden (Marktkonformität).

Diese beiden Grundsätze müssen auch bei Eingriffen in die Wirtschaftsstruktur beachtet werden. Wo immer möglich, sollten regelbasierte Märkte zur Koordinierung dienen. Die Eingriffe des Staates dürfen die Prozesse auf diesen Märkten nicht unnötigerweise beeinflussen, indem sie etwa staatlichen Unternehmen Vorteile einräumen.

## Kriterien für Staatseingriffe durch Beteiligungen

Die Entscheidung, ob überhaupt ein staatlicher Eingriff notwendig ist, sollte auf Basis feststehender Kriterien erfolgen. Diese Kriterien sollten idealerweise bereits ex ante im politischen Prozess entwickelt worden sein und vor einem Eingriff feststehen. Dadurch kann unter anderem der Eindruck vermieden werden, dass Beteiligungen und damit die Verwendung von Steuergeldern willkürlich erfolgen oder ein erratischer Umgang mit Steuergeldern staatliches Handeln unvorhersehbar erscheinen lässt. Eine Zufälligkeit staatlichen Handelns kann in der Krise fatal wirken, wenn unklar ist, wer überhaupt gerettet wird. Es ist daher wichtig, vor – beziehungsweise während – des staatlichen Eingriffs dessen Ziele festzulegen und zu kommunizieren, um Wirtschaft und Gesellschaft bei einer solchen Entscheidung zu informieren und einzubeziehen. Mögliche Ziele der Staatsbeteiligung gerade während der Corona-Pandemie könnten sein:

- Stabilisierung in der Krise: Die Krise veranlasst den Staat, Unternehmen zu stabilisieren, die allein durch die Pandemie in ihrer Existenz bedroht wären. Das Erzielen von Gewinn und das Vermeiden von Verlusten sind dabei keine expliziten Ziele; ein Ausstieg darf beispielsweise nicht davon abhängig gemacht werden, dass frühere Börsenkurse wieder erreicht wurden.

- Analog zur Regulierung bei ausländischen Unternehmensübernahmen (§55 der AWV – Außenwirtschaftsverordnung) kann ein Staatseingriff zur Aufrechterhaltung der öffentlichen Ordnung oder Sicherheit der Bundesrepublik gerechtfertigt sein. Denkbar ist in diesem Zusammenhang unter anderem der Erhalt infrastrukturell unverzichtbarer Unternehmen, Produzenten wichtiger Software (z. B. für Kraftwerksleittechnik oder Telekommunikationsnetze) und Produzenten von wichtigen Gütern sowie Dienstleistungen (Medikamente oder Rüstungsgüter). In diesen Fällen dienen die Unternehmen der Verfolgung politischer oder strategischer Ziele wie der Schaffung von Versorgungssicherheit und der Schaffung sowie Erhaltung von Know-how, wodurch staatliche Unterstützung gerechtfertigt ist.

- Verhinderung von Dominoeffekten: Unternehmen wie Großbanken oder Netzbetreiber, die strukturell stark vernetzt sind und deren kurzfristiges, unkontrolliertes Ausscheiden erhebliche negative Auswirkungen auf andere Unternehmen oder die Gesellschaft hätte, können ebenfalls mittels Staatsbeteiligung unterstützt werden. Dies sollte dazu dienen, einen unkontrollierten Zusammenbruch zu verhindern und das Ausscheiden sowie eine Erneuerung des Unternehmens in geregelten Bahnen zu ermöglichen.

Zur Umsetzung dieser Ziele benötigt es klare Kriterien, die ex ante formuliert sein sollten. Abbildung 2 fasst die wichtigsten Kriterien für die Entscheidung über eine Staatsbeteiligung in einem Prüfschema zusammen. Für das Eingehen einer staatlichen Beteiligung sollten folgende Kriterien – in der Regel kumulativ – erfüllt sein:

- Es muss ein plötzlicher, unvorhergesehener Markteinbruch vorliegen, der ein zuvor gesundes Unternehmen in eine nicht selbst verschuldete, existenzgefährdende Krise gestürzt hat.

- Unternehmen, an denen der Staat eine Beteiligung eingeht, müssen eine Relevanz für den Markt oder die öffentliche Ordnung und Sicherheit des Landes besitzen. Hierfür sind insbesondere zwei Faktoren entscheidend:

Abbildung 2
**Entscheidung über eine staatliche Beteiligung**

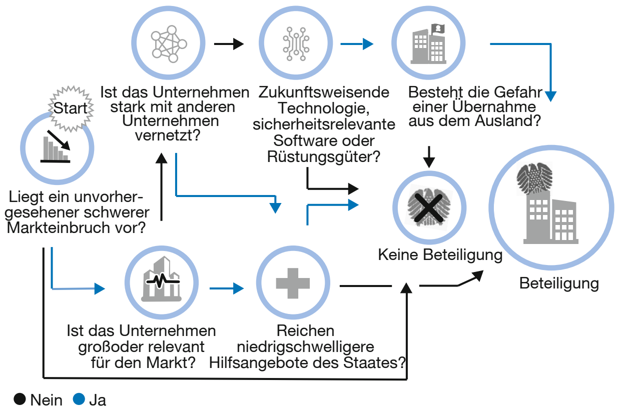

Quelle: eigene Darstellung.

- Das Unternehmen besitzt eine erhebliche Größe oder ist stark mit anderen Unternehmen vernetzt, was sich bei einer kurzfristigen Insolvenz negativ auf den Gesamtmarkt (Kunden/Zulieferer) bzw. viele andere Unternehmen auswirkt, weil beispielsweise eine wichtige Infrastruktur nicht wie gewohnt zur Verfügung steht (Dominoeffekt). Hierunter fällt auch das Kriterium der Netzwerkrelevanz, das etwa bei der Beteiligung an der Lufthansa als nationalem Carrier mit globalem Streckennetz, das die international stark verflochtene deutsche Wirtschaft benötigt, zur Geltung kam. Größe und Arbeitsmarktrelevanz allein bilden keine ausreichenden Kriterien, da andernfalls die Gefahr besteht, durch Staatsbeteiligungen den Strukturwandel zu behindern.

- Das Unternehmen verfügt über eine zukunftsweisende Technologie, entwickelt sicherheitsrelevante Software oder Rüstungsgüter. Ohne die Staatsbeteiligung sollte die Gefahr eines Ausscheidens des Unternehmens oder einer ausländischen Übernahme bestehen. Als Definition relevanter Technologiefelder könnten das Außenwirtschaftsgesetz und als erste Näherung die in der Außenwirtschaftsverordnung (BMWi, 2020) definierten Branchen verwendet werden. Zumindest theoretisch fällt auch die Beteiligung am Biotechunternehmen CureVac in diese Kategorie. Allerdings blieb unklar, ob ein derartiger Übernahmewunsch tatsächlich vorlag, die Impfstoffentwicklung des Unternehmens von Erfolg gekrönt sein wird und warum ebenso vielversprechende Wettbewerber nicht unterstützt wurden. Auch hier sind niedrigschwelligere Alternativen wie ein Verbot der ausländischen Übernahme und Hilfen bei der Organisation inländischer Beteiligungen aus der Privatwirtschaft vorzuziehen.

- Es besteht eine Lücke im Portfolio staatlicher Hilfsangebote. Als weiteres Kriterium sollte geprüft werden, ob alternative Hilfsangebote des Staates unterhalb des schweren Markteingriffs, den eine Beteiligung bewirkt, nicht ausreichen. Hierzu zählen in der aktuellen Corona-Krise etwa vergünstigte Kredite und erweiterte Kurzarbeitsregelungen.

Eine staatliche Beteiligung oder gar vollständige Übernahme ist wohl die unmittelbarste Form eines staatlichen Eingriffs in ein Unternehmen. Jedoch handelt es sich dabei nur um eine mögliche Form eines Wirtschaftseingriffs. Neben einer Staatsbeteiligung stehen beispielsweise vertiefte Kooperationen sowie das Mittel einer moderierten Fusion zur Verfügung. Eine engere Kooperation kann mehrere Unternehmen in einem betroffenen Markt stabilisieren und helfen, Herausforderungen zu überwinden, weshalb diese Maßnahme staatlicherseits angeregt werden sollte. In der gegenwärtigen Pandemie werden Kooperationen von Kartellbehörden relativ großzügig gehandhabt (European Competition Network, [ECN], 2020). Die Fusion zweier Wettbewerber ist eine langfristige und zumeist irreversible Marktveränderung. Marktkonformität wird damit zu einem wichtigen Kriterium. Das heißt, es sollte nicht zu Fusionen kommen, die nicht durch unternehmerische Entscheidungen zustande gekommen wären, oder die unternehmerisch nicht sinnvoll sind.

Eine staatliche Beteiligung hingegen, insbesondere wenn sie als stille Minderheitsbeteiligung erfolgt, hat einen variablen Zeithorizont und kann, falls die auslösende Krise eher kurzfristig ist, wieder rückgängig gemacht werden. Die Corona-Pandemie ist eine (hoffentlich) vorübergehende Krise und die Kosten für eine staatliche Beteiligung können gerechtfertigt sein, weil langfristige Kosten beispielsweise durch die Verringerung von Wettbewerb bei Marktaustritten oder die Reduktion von Innovationen bei Fusionen vermieden werden. Somit sollte als Reaktion auf die Pandemie zunächst eine Beteiligung einer Fusion vorgezogen werden. Eine Fusion kann vorzugswürdig sein, wenn beispielsweise infolge eines langfristigen Strukturwandels die Nachfrage nachhaltig sinkt oder Überkapazitäten bestehen. In diesem Fall kann der Strukturwandel gezielt gesteuert werden. Ein Beispiel hierfür ist die Gründung der Ruhrkohle AG (RAG) 1968 (Klute, 2019), der allerdings jahrzehntelange problematische Subventionen für die Steinkohleförderung folgten.

## Rahmenbedingungen nicht aus den Augen verlieren

Neben Staatsbeteiligungen oder vermittelten Fusionen stellt auch die Verbesserung relevanter Rahmenbedingungen ein politisches Handlungsfeld dar, das nicht vernachlässigt werden darf. Beispielsweise können die Bedingungen für alle Unternehmen durch zügige Genehmigungsverfahren oder ein verständliches Steuerrecht mit international konkurrenzfähigen Steuersätzen verbessert werden, wodurch Innovationsaktivitäten angeregt werden und der Wettbewerb als Selektionsmechanismus seine volle Wirkung entfalten kann. Es setzt sich eher das Unternehmen durch, das die Kundenbedürfnisse am besten erfüllt. Mittels Verbesserung der Rahmenbedingungen kann auch auf identifizierte Fehlentwicklungen wie die Abwanderung oder den Verkauf innovativer Unternehmen reagiert werden.

Eine Stärkung der Rahmenbedingungen sollte ebenfalls die Verbesserung der Finanzierungsmöglichkeiten für junge Unternehmen, etwa im Venture-Capital-Bereich, beinhalten. Es ist zu beachten, dass es bereits eine mehr oder weniger umfangreiche Forschungs- und Entwicklungs- (FuE) sowie Technologieförderung gibt, die vielen innovativen Unternehmen zugutekommt. Die Technologiefeld- und projektbezogene FuE-Förderung wurde erst zum Jahresanfang 2020 um eine steuerliche Forschungsförderung erweitert (Deutscher Bundestag, 2019), die jedoch restriktiver ausgestaltet wurde, als es Empfehlungen aus der Wissenschaft und der Wirtschaft vorsahen. Möglicherweise sind die knappen staatlichen Mittel in einer großzügigeren steuerlichen FuE-Förderung, die allen innovativen Unternehmen offensteht, zielführender einzusetzen als in Beteiligungen an einzelnen Hochtechnologieunternehmen. Auch Rahmenbedingungen etwa im Bereich der steuerlichen Verlustverrechnung sind in Deutschland restriktiver ausgestaltet als in anderen hoch entwickelten Ländern, mit denen Deutschland im Standortwettbewerb um technologieintensive Start-ups steht (LM Law Rechtsanwaltsgesellschaft mbH, o.J.; Röhl, 2014).

## Fazit

Die Corona-Pandemie ist ein historischer Schock für die Wirtschaft und die gesamte Gesellschaft in Deutschland und weltweit. Betroffen ist neben der Industrie aufgrund des Lockdowns und anhaltender Kontaktbeschränkungen vor allem der Dienstleistungsbereich, der in vielen entwickelten Ländern für über 70 % und in Deutschland für mehr als 68 % der gesamten Wirtschaftsleistung steht (United Nations, 2019). Die erneute Zunahme der Fallzahlen dürfte die wirtschaftliche Erholung verzögern und die Krise verlängern.

Schwere ökonomische Schocks wie diese bergen zwar auch das Potenzial, den Strukturwandel durch digitale Anwendungen und Geschäftsmodelle zu beschleunigen und damit Produktivitätsfortschritte und ein erhöhtes Wachstum zu ermöglichen. Sie können jedoch zu einer tiefgreifenden Krise bis hin zur Depression führen, der auch gesunde Unternehmen zum Opfer fallen und die die Basis für neues Wachstum zerstört. Eine Beschleunigung der Digitalisierung kann dabei die Marktmacht großer digitaler Plattformen erhöhen (Monopolkommission, 2020; Demary, 2020). Der Staat wiederum kann durch sein Handeln die ökonomischen Auswirkungen auf die Konsumenten und Unternehmen abmildern und eine Krisenverschärfung verhindern. Ein staatlicher Eingriff kann zudem gerechtfertigt sein, wenn der Auslöser der Krise wie aktuell kein ökonomischer ist. Zu weitreichende staatliche Eingriffe, insbesondere wenn sie ineffiziente Strukturen konservieren, Verzerrungen des Marktes durch Staatseingriffe und Staatsbeteiligungen erhöhen sowie die knappen Ressourcen des Staates überlasten, können allerdings einem wirtschaftlichen Aufschwung die Basis entziehen und langfristig negative Struktureffekte bewirken.

Deshalb ist bei staatlichen Maßnahmen auf Marktkonformität zu achten. Dies beinhaltet bei Eingriffen in die Wirtschaftsstruktur, etwa mittels staatlich angeregter Fusionen und Beteiligungen, dass im Rahmen der Krise keine Strukturen geschaffen werden dürfen, die sich nicht selbst auf dem Markt gebildet hätten oder die am Wettbewerbsrecht gescheitert wären. Bei direkten Staatsbeteiligungen gilt es zudem, die Entscheidungen im Unternehmen nicht politisch zu beeinflussen, den Wettbewerb nicht zu verzerren und den Ausstieg des Staates nach der Krise nicht aus dem Blick zu verlieren. Die Kriterien für Entscheidungen über eine Staatsbeteiligung sollten ex ante festgelegt werden und transparent sein. Neben direkten oder indirekten Eingriffen des Staates in die Wirtschaftsstruktur stellt die Verbesserung der Rahmenbedingungen ebenfalls ein wichtiges Handlungsfeld dar. Hier zeichnete sich für Deutschland im Bereich der Infrastruktur, der Steuerpolitik und des Planungs- und Genehmigungsrechts schon vor der Corona-Krise Handlungsbedarf ab.
